# Editorial: Growth factors and stem cells intervention in injury repair and regeneration of spinal cord and peripheral nerve

**DOI:** 10.3389/fphar.2022.1115470

**Published:** 2023-01-09

**Authors:** Min Chen, Juan Zhao, Xiong Cai, Sipin Zhu

**Affiliations:** Department of Orthopaedics, The Second Affiliated Hospital and Yuying Children’s Hospital of Wenzhou Medical University, Wenzhou, Zhejiang, China

**Keywords:** regeneration, growth factor, stem cell, spinal cord injury, peripheral nerve injury

Injuries of the central nervous system (CNS), including stroke, spinal cord injury, and traumatic brain injury, can cause serious and permanent disability due to limited regeneration ability of the CNS. Unlike the central nervous system, peripheral nerves have the ability to regenerate lost axons after injury. However, axonal regeneration does not equate to full restoration of function. Thus, despite the peripheral nervous system’s intrinsic ability to regenerate, many patients experience incomplete functional recovery. Therefore, the repair of nervous system is still an urgent clinical problem.

Growth factors are a class of peptide cytokines that regulate cell growth and proliferation. There are many kinds of growth factors such as fibroblast growth factor (FGF), epidermal growth factor (EGF), insulin-like growth factor (IGF), nerve growth factor (NGF), platelet-derived growth factor (PDGF), transforming growth factor (TGF), etc. Thus, they are involved in the regulation of histomorphological changes and play a regulatory role in cell differentiation, migration and functional activities.

This Research Topic gathers original research and review papers on the nerve repair based on growth factors and stem cells. This Research Topic of papers sheds light on the interaction between the components of the nerve injury microenvironment and the intracellular signaling, ranging from basic research to clinical translational studies.

The seven accepted articles consist of four original research articles, two systematic review and one review, which demonstrated different targets and signaling pathways for central and peripheral nervous system repair therapy.

Two original research articles elucidated that growth factors are involved in alleviating neural diseases *via* different mechanisms. Talifu et al. found that the overexpression of IGF-1 and NT3 can promote the functional recovery and alleviate spasticity after spinal cord injury through the increased expression of KCC2 in the motor neuron membrane and levels of the 5-HT2A/2C receptor. It related to the neuroprotective effect of nerve growth factors, the reorganization of neural circuits in the spinal cord, and the upregulation of the expression of related channel proteins. The recovery may be related to the neuroprotective effect of nerve growth factors, the reorganization of neural circuits in the spinal cord, and the upregulation of the expression of related channel proteins Talifu et al. Zhang et al. explored a new type of growth factor administration, which combined basic fibroblast growth factor (bFGF) with sodium alginate hydrogel (called ALG-bFGF) to the sustained release of bFGF. They used SCI mice models, oxygen glucose deprivation (OGD)–stimulated human brain microvascular endothelial cells (HBMECs), and specific inhibitors, the work demonstrates that ALG-bFGF hydrogel inhibits autophagy activation by regulation of the PI3K/Akt/FOXO1/KLF4 pathway, which contributes to restoration of BSCB integrity and locomotor function recovery after SCI Zhang et al.

The other two original research articles focused on effect and mechanism of traditional Chinese medicine (TCM) components on nerve repair. Huang et al. explored the neuroprotective effect of rehmannia glutinosa, which has been used in TCM to treat aging-related conditions including dementia and senile diseases. Catalpa is one of the main pharmacological components of rehmannia glutinosa, which has the best cell protective effect. The SCI rats fed with rehmannia decoction for 30 days showed a slight improvement in hindlimb coordination and toe dragging. The result shown that after treatment with CAT, the protein levels related to ER stress were all downregulated and the levels such as apoptosis promoting protein Bax and Caspase3 were inhibited, whereas the level of apoptosis inhibiting protein Bcl-2 was increased. It was further demonstrated that CAT reduced apoptosis by inhibiting Caspase3/Bax/Bcl-2 pathway. The increased expression of NeuN and large expression of GAP43 and MAP-2 suggested that neurons were undergoing further recovery Huang et al. Zhang et al. used radix astragalus polysaccharide (RAP), the main pharmacological component of astragalus promote sciatic nerve regeneration *via* accelerating angiogenesis. RAP promoted functional recovery after PNI, delayed target organ atrophy, and mediates regeneration and remyelination of axons. Furthermore, the positive effect of RAP on nerve regeneration was associated with angiogenesis that may be associated with activation of the AKT/eNOS signaling pathway Zhang et al. Overall, these researches suggested that TCM may be a potentially effective therapeutic agent for nerve repair.

The two systematic reviews discussed the effect of stem cells on SCI recovery. Yang et al. summarized the EVs derived from miRNA-modified MSCs for the treatment of SCI included data from 13 preclinical trials among a total of 396 rats. This review indicated that miRNA-loaded EVs may be most effective for the treatment of contusion models at the early, middle, and late stages Yang et al. Therefore, miRNA-loaded EVs have tremendous therapeutic potential within SCI. Shang et al. collected the animal studies of stem cell therapy for SCI at home and abroad, explore the real effects of different stem cell therapies. This review comprehensively analyzed the efficacy of transplantation of four types of stem cells at different time points after spinal cord injury and found that UCMSCs may be the most effective stem cells for the treatment of SCI in the first week after stem cell transplantation, while ADMSCs may be the most effective stem cells in the third, fifth, and eighth weeks after stem cell transplantation. In conclusion, high-dose stem cell transplantation into the focal center during the subacute phase can significantly improve the efficacy of stem cell transplantation. The only review summarized the involvement of TGF-β in peripheral nerve regeneration and discuss the possible underlying mechanisms and the application of TGF-β in the experimental treatment of PNI (Ye et al.) As a pleiotropic cytokine with neuroprotective functions, TGF-β is a promising target for promoting nerve regeneration and recovery after PNI. Hou et al. introduced the role of TGFs in peripheral nerve repair, including promoting Schwann cell reprogramming, removing myelin debris, repairing blood nerve barrier and anti-inflammation.

In summary, both the research articles and reviews in this Research Topic are an excellent source of information about the regeneration of spinal cord and peripheral nerve injury.

